# Multivariate analysis reveals differentially expressed genes among distinct subtypes of diffuse astrocytic gliomas: diagnostic implications

**DOI:** 10.1038/s41598-020-67743-7

**Published:** 2020-07-09

**Authors:** Nerea González-García, Ana Belén Nieto-Librero, Ana Luisa Vital, Herminio José Tao, María González-Tablas, Álvaro Otero, Purificación Galindo-Villardón, Alberto Orfao, María Dolores Tabernero

**Affiliations:** 1 Department of Statistics, University of Salamanca, Salamanca, Spain; 2 Instituto de Investigación biomédica de Salamanca, IBSAL- University Hospital of Salamanca, Salamanca, Spain; 3 Centre for Neuroscience and Cell Biology and Faculty of Pharmacy, University of Coimbra, Coimbra, Portugal; 4 Neurosurgery Service, University Hospital of Coimbra, Coimbra, Portugal; 5 Centre for Cancer Research (CIC-IBMCC; CSIC/USAL; IBSAL) and Department of Medicine, University of Salamanca, Salamanca, Spain; 6 Biomedical Research Networking Centre on Cancer–CIBERONC (CB16/12/00400),, Institute of Health Carlos III, Madrid, Spain; 7 Instituto de Estudios de Ciencias de La Salud de Castilla y León (IECSCYL-IBSAL), Salamanca, Spain

**Keywords:** Cancer, Computational biology and bioinformatics, Genetics, Neuroscience, Medical research, Oncology

## Abstract

Diagnosis and classification of gliomas mostly relies on histopathology and a few genetic markers. Here we interrogated microarray gene expression profiles (GEP) of 268 diffuse astrocytic gliomas—33 diffuse astrocytomas (DA), 52 anaplastic astrocytomas (AA) and 183 primary glioblastoma (GBM)—based on multivariate analysis, to identify discriminatory GEP that might support precise histopathological tumor stratification, particularly among inconclusive cases with II–III grade diagnosed, which have different prognosis and treatment strategies. Microarrays based GEP was analyzed on 155 diffuse astrocytic gliomas (discovery cohort) and validated in another 113 tumors (validation set) via sequential univariate analysis (pairwise comparison) for discriminatory gene selection, followed by nonnegative matrix factorization and canonical biplot for identification of discriminatory GEP among the distinct histological tumor subtypes. GEP data analysis identified a set of 27 genes capable of differentiating among distinct subtypes of gliomas that might support current histological classification. DA + AA showed similar molecular profiles with only a few discriminatory genes overexpressed (*FSTL5* and *SFRP2*) and underexpressed (*XIST, TOP2A* and *SHOX2*) in DA vs AA and GBM. Compared to DA + AA, GBM displayed underexpression of *ETNPPL, SH3GL2, GABRG2, SPX, DPP10, GABRB2* and *CNTN3* and overexpression of *CHI3L1, IGFBP3, COL1A1* and *VEGFA*, among other differentially expressed genes.

## Introduction

Diffuse gliomas comprise a variety of tumor entities of different cell lineages and histopathological features which are classified into distinct subtypes by the World Health Organization (WHO)^1,2^, from which astrocytic lineage tumors (i.e. astrocytomas and glioblastomas) are by far the most common (around 90%)^3^. Relevant histological and immunohistochemical features together with the presence of codeletion of chromosome 1p/19q and isocitrate dehydrogenase 1 (*IDH1*) gene mutation, are currently used for the differential diagnosis between oligodendroglial tumors and diffuse astrocytomas^1,4^. However, further differential diagnoses among the distinct subtypes of diffuse astrocytomas might be challenging and they might even lead to inconclusive results, particularly among grade II and III tumors. For this purpose molecular characteristics of these tumors have been recurrently investigated. However, while genetic alterations are found in the majority of tumors, they are not entirely specific, and thereby they are not considered in the current WHO-2016 classification of gliomas. Thus, astrocytic gliomas are currently divided in non-diffuse (pilocytic astrocytoma grade-I, PA; WHO-I) and diffuse tumors based on conventional histopathology. The latter tumors were further divided on histopathological grounds into three grades of malignancy associated with distinct median survival rates (range: from 1 to > 10 years)^5,6^: WHO grade-II diffuse astrocytoma (DA), WHO grade-III anaplastic astrocytoma (AA) and WHO grade-IV glioblastoma (GBM). Of note, DA might evolve to AA, and AA might transform to GBM^7,8^. Because of this, GBM is further subdivided into primary *de novo* GBM (pGBM) and secondary GBM (sGBM) resulting from progression of a prior lower grade astrocytic tumor (e.g. AA)^7^.

At present, the *IDH1*-mutation together with codeletion of chromosomes 1p/19q have become a major criterion for the differential diagnosis between oligodendrocytic and oligoastrocytic tumors vs diffuse astrocytic gliomas^1,2^. However, DA and AA also show a variable frequency of *IDH*-mutation, while this mutation is absent in the great majority of GBM^9^, which limits its diagnostic utility among diffuse astrocytic tumors. Other genetic markers that have been associated with specific subtypes of astrocytomas and diffuse astrocytic tumors could be useful for glioma classification^10,11^, include gains and losses of specific chromosomal regions together with mutations of the *EGFR, MDM4, PTEN, PDGFRA* and *CDKN2A* genes, but they are not considered in the WHO-2016 classification^2^. Altogether, this highlights the need for deeper genomic analysis of astrocytic tumors to gain further insight in those gene profiles that might help to unequivocally distinguish among the different subtypes of astrocytic tumors and support the differential diagnosis and subclassification of diffuse gliomas, particularly in cases^12,13^ with an inconclusive histopathological diagnosis.

Despite the expression levels of specific genes, such as *CHI3L1* and *TOP2A, w*hich have been related to necrosis in GBM^14,15^, and *IGFBP2* and *VEGFA* involved in tumor progression^12^, mRNA-based gene expression profiling (GEP) has frequently shown discrepant results in gliomas, hampering application of GEP in clinical practice. To a certain extent such discrepancies are due to: (i) analysis of small patient cohorts^16^, (ii) focused on individual tumor types such as GBM^17^, together with (iii) the use of different microarray platforms, and/or iv) diverse mathematical approaches and multivariate data analysis algorithms^12^. As an example, principal component analysis (PCA)^18^ has been frequently used for GEP analysis, despite its limitations for the analysis of high-dimensional databases that contain a number of variables that significantly exceeds the number of tumors. For such situations, variable selection techniques together with other matrix factorization algorithms, such as nonnegative matrix factorization (NMF)^19^, have been proposed for the discovery of clusters that might gather important biological information, as recently demonstrated in pancreatic cancer^20^.

In this study we aimed at identifying a panel of informative genes for subclassification of a large series of 268 astrocytic diffuse gliomas into their DA, AA and GBM subtypes based on GEP data analyzed with combination of low-rank matrix decompositions, as CUR decomposition^21^, followed by NMF^19^ and canonical biplot^22^. A panel of 27 discriminatory genes were finally identified that efficiently differentiate among the three subtypes of diffuse astrocytic tumors.

## Results

**Figure 1:**
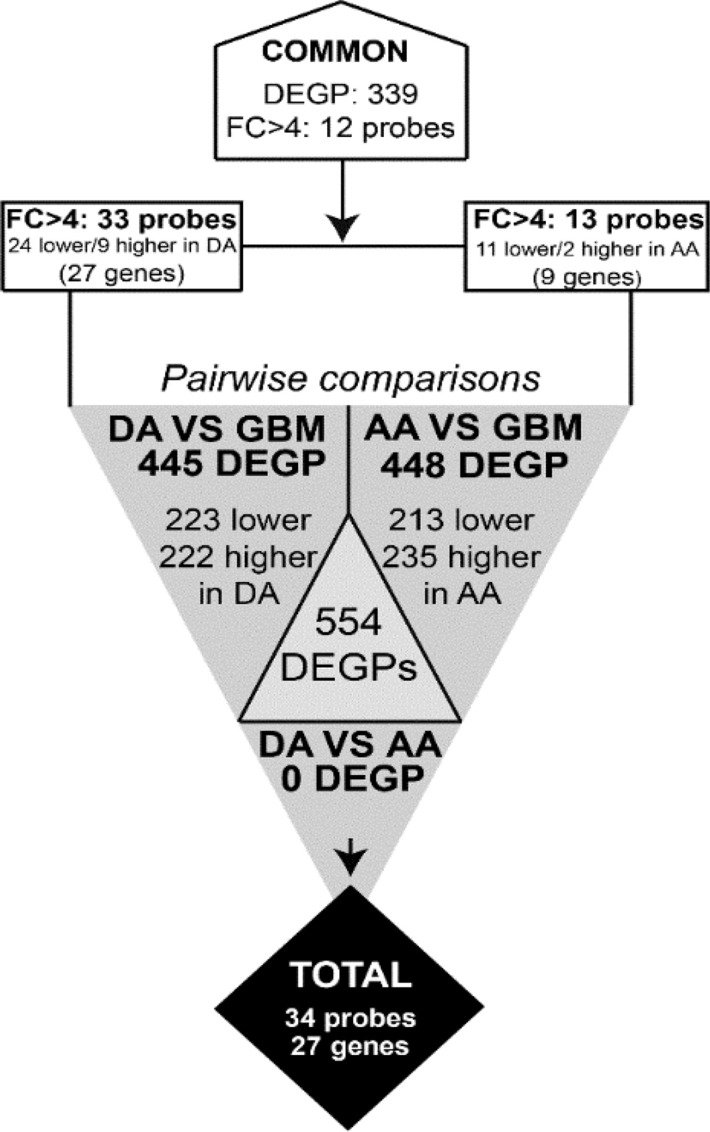
Number of differentially expressed gene probes in samples corresponding to distinct tumor subtypes from the discovery cohort. Differentially expressed probes were identified by pairwise comparisons based on p-values < 0.05. The number of overexpressed and underexpressed probes in DA and AA versus GBM are shown. DEGP, differentially expressed gene probes; DA, diffuse astrocytoma; AA, anaplastic astrocytoma; GBM, glioblastoma; FC, fold-change in gene expression levels.

Pairwise comparisons of gene expression data from the discovery cohort showed > 800 differentially expressed gene probes among the three subtypes of diffuse astrocytic tumors analyzed (Fig. 1) with significant adjusted P-values. Despite this, no gene probe showed significant differences in gene expression (discriminatory) levels between DA and AA tumors. In contrast, 445 gene probes were differentially expressed (P < 0.05) between DA and GBM (222 were underexpressed in GBM and 223 overexpressed) and 448 gene probes where distinctly expressed (P < 0.05) in AA vs GBM (235 underexpressed in GBM and 213 overexpressed probes); 339 probes corresponding to 283 genes were differentially expressed in common in the two above comparisons (Fig. 1). Comparison between DA vs GBM revealed 33/445 differentially expressed gene probes, corresponding to 27 distinct genes, with fold-change (FC) value differences (vs mean probe intensity) of *FC* ≥ 4 (Table 1). These included 9/27 genes with increased expression values in DA vs GBM (*ETNPPL, FSTL5, SFRP2, SH3GL2, CNTN3, SPX, GABRG2, GABRB2, DPP10*) and 18/27 genes with higher expression in GBM vs DA (*CHI3L1, COL1A1, COL3A1, POSTN, COL1A2, IGF2BP3, NNMT, SHOX2, XIST, HS3ST3B1, PTX3, VEGFA, IBSP, TOP2A, LOX, IGFBP3, ANXA1, PDPN)* (Fig. 1; Table 2). Similarly, the comparison between AA and GBM revealed 13/448 differentially expressed gene probes corresponding to 9 genes to display *FC* ≥ 4 (vs mean probe intensity). Once compared to GBM, AA showed overexpression of two gene probes (*CNTN3* and *ETNPPL* genes) and underexpression of another 11 gene probes corresponding to 7 genes: *CHI3L1, COL1A1, COL3A1, POSTN, NNMT, PTX3, COL1A2* (Fig. 1; Table 1).

**Table 1:** Gene probes that showed significant discriminatory power -fold-change (FC) > 4- among distinct histopathological subtypes of gliomas as identified in pairwise comparisons (n = 34 probes corresponding to 27 different genes).

Differentially expressed probes		Pairwise comparison^a^	
Probe set	Gene symbol	DA vs GBM	AA vs GBM
201012_at	*ANXA1*	4.07	
209396_s_at	*CHI3L1*	9.14	5.82
209395_at		9.35	5.9
229831_at	*CNTN3*	4.79	4.45
1556499_s_at	*COL1A1*	6.57	4.47
202310_s_at			5.76
202404_s_at	*COL1A2*	5.78	4.5
201852_x_at	*COL3A1*	5.73	5.04
215076_s_at		6.34	5.5
211161_s_at		6.56	5.72
228598_at	*DPP10*	4.07	
221008_s_at	*ETNPPL*	8.73	6.3
232010_at	*FSTL5*	6.69	
242344_at	*GABRB2*	4.17	
1568612_at	*GABRG2*	4.3	
227361_at	*HS3ST3B1*	4.97	
236028_at	*IBSP*	4.49	
203819_s_at	*IGF2BP3*	5.2	
203820_s_at		5.4	
210095_s_at	*IGFBP3*	4.25	
215446_s_at	*LOX*	4.34	
202237_at	*NNMT*	5.3	5.06
221898_at	*PDPN*	4.06	
210809_s_at	*POSTN*	6.24	5.63
206157_at	*PTX3*	4.87	4.98
223122_s_at	*SFRP2*	6.58	
205751_at	*SH3GL2*	4.88	
210135_s_at	*SHOX2*	5.29	
229778_at	*SPX*	4.67	
201291_s_at	*TOP2A*	4.43	
211527_x_at	*VEGFA*	4.58	
224590_at	*XIST*	4.04	
221728_x_at		4.07	
224588_at		5.19	

Results expressed as fold-change (FC) values for pairwise comparisons.

*DA* diffuse astrocytoma, *AA* anaplastic astrocytoma, *GBM* glioblastoma multiforme.

**Table 2:** Gene expression levels for the 27 genes differentially expressed among the three histopathological subtypes of diffuse astrocytic gliomas analyzed.

Gene	Chromosomal location	Gene expression values in glioma subtypes		
		DA (n = 19)	AA (n = 28)	GBM (n = 108)
*GABRG2*	5q34	7.72	7.3	5.62
*CNTN3*	3p12.3	7.75	7.64	5.49
*GABRB2*	5q34	7.9	7.79	5.84
*SPX*	12p12.1	8.07	7.77	5.84
*DPP10*	2q14.1	8.09	7.86	6.07
*FSTL5*	4q32.3	8.27	7.06	5.53
*SH3GL2*	9p22	9.75	9.16	7.47
*SFRP2*	4q31.3	9.87	8.5	7.15
*ETNPPL*	4q25	10.31	9.84	7.19
*SHOX2*	3q25.32	4.83	5.82	7.23
*IBSP*	4q21.1	5.04	5.22	7.2
*IGF2BP3*	7p11	5.25	6.06	7.66
*LOX*	5q23.2	5.37	5.65	7.48
*HS3ST3B1*	17p12	5.49	5.98	7.81
*XIST*	Xq13.2	5.5	8.33	7.64
*PTX3*	3q25	6.29	6.26	8.57
*POSTN*	13q13.3	6.46	6.61	9.1
*TOP2A*	17q21.2	6.49	7.44	8.64
*VEGFA*	6p12	6.94	7.26	9.13
*COL3A1*	2q31	7	7.2	9.64
*NNMT*	11q23.1	7.25	7.31	9.65
*COL1A2*	7q22.1	7.44	7.8	9.97
*COL1A1*	17q21.33	7.47	7.45	9.8
*PDPN*	1p36.21	7.89	7.98	9.91
*CHI3L1*	1q32.1	8.65	9.31	11.86
*IGFBP3*	7p12.3	8.73	9.43	10.81
*ANXA1*	9q21.13	9.09	9.65	11.12

Results expressed as mean (SD) values.

*DA* diffuse astrocytoma, *AA* anaplastic astrocytoma, *GBM* glioblastoma multiforme.

Based on the list of gene probes identified with both approaches, a total of 27 differentially expressed genes were selected for subsequent multivariate analyses (Table 2 and Supplementary Table S1). More detailed analysis of those 27 genes found that *XIST* was the only gene with the most clearly different expression profile between DA and AA tumors (Table 2). Among the other 26 genes, *ETNPPL, SFRP2, SH3GL2, FSTL5, DPP10, SPX, GABRB2, CNTN3* and *GABRG2* displayed greater expression levels in DA + AA vs GBM. Of these latter 9 genes, 3 are coded in chromosome 4 (*ETNPPL, SFRP2* and *FSTL5*), 2 in chromosome 5 (*GABRB2*, *GABRG2*), 1 in chromosome 2 (*DPP10*), 1 in chromosome 3 (*CNTN3*), 1 in chromosome 9 (*SH3GL2*) and the *SPX* gene is coded in chromosome 12. Likewise, another 17 genes, including the *CHI3L1, COL1A1, IGFBP3* genes, were overexpressed in GBM vs both DA + AA. More than half of these later genes (10/17 genes) are coded in a total of only 4 chromosomes, including chromosomes 7 (*COL1A2, IGF2BP3, IGFBP3*), 17 (*COL1A1*, *HS3ST3B1*, *TOP2A*), 1 (*CHI3L1, PDPN*) and 3 (*PTX3, SHOX2*). The remaining 7 genes are coded each in a distinct chromosome, e.g. chromosomes 2 (*COL3A1*), 4 (*IBSP*), 5 (*LOX*), 6 (*VEGFA*), 9 (ANXA1), 11 (*NNMT*) and 13 (*POSTN*). Interestingly, genes coded in the long arm of chromosome 2q, 4q and 5q appeared to be relevant in all group comparisons (Table 2).

**Figure 2:**
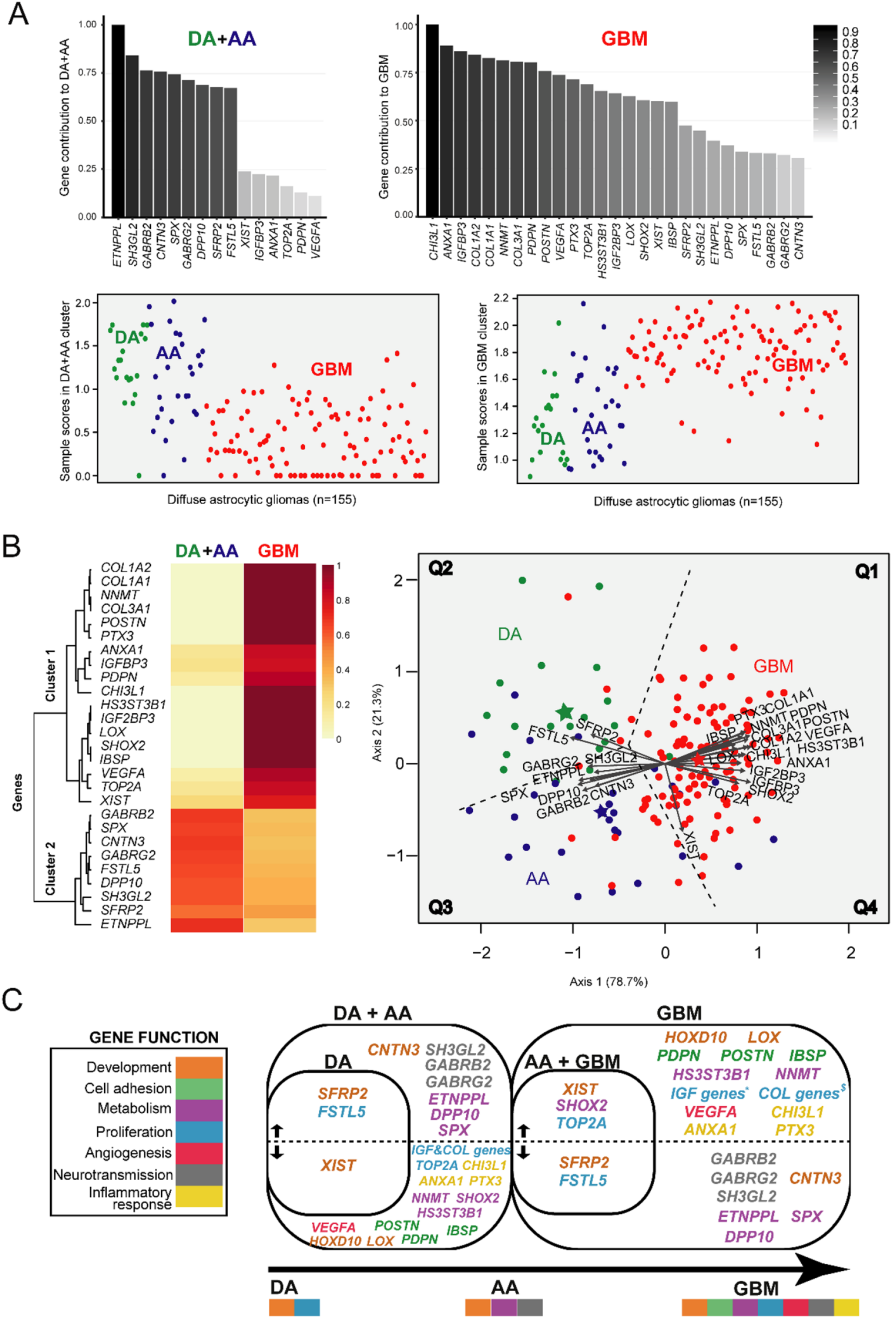
Multidimensional relationship between the 27 genes selected and the histological subtypes of diffuse astrocytic tumors. NMF analysis revealed two gene clusters based on the coordinates of distinct sets of genes to each cluster shown in the bar charts and heatmap (Upper graphics in panel **A**, left graphic in panel **B**). Scores obtained by NMF (corresponding to the grade of membership of each sample to the defined clusters) detected two major subsets of gliomas among DA (dipicted green), AA (blue) and GBM (red) (panel **A**): one group consisting mainly of GBM and a few samples of AA cases and the other group included both DA and AA. The canonical biplot representation is shown in the right of Panel **B**, where the mean values of each subgroup of astrocytic tumors is plotted with a star colored (individual DA, AA and GBM tumors are labeled as green, blue and red points) and the discriminatory genes are plotted as vectors. The discriminatory genes found to distinguish among the three WHO subtypes of diffuse astrocytic tumors are listed in panel **C** in a color code defined by their known functions. R software for statistical computing and graphics (v3.5.2) was used. DA, diffuse astrocytoma; AA, anaplastic astrocytoma; GBM, glioblastoma.

NMF was subsequently applied to the discovery cohort of 155 diffuse astrocytic glioma GEP data about of the 27 genes previously selected, in order to further establish the relevance of the selected genes to classify the 3 subtypes of diffuse astrocytic gliomas. Two different clusters (corresponding to DA + AA and GBM) were detected with NMF, DA and AA tumors being graphically visualized as a single cluster (Fig. 2A). Once we investigated the relevance of each gene to the formation of both clusters (Fig. 2A) we confirmed that the *ETNPPL, SH3GL2, GABRB2, CNTN3, SPX, GABRG2, DPP10, SFRP2, FSTL5* genes where those most contributing to the DA + AA cluster, followed by a few genes displaying a lower contribution (*XIST, IGFBP3, ANXA1, TOP2A, PDPN* and *VEGFA)* (Fig. 2B)*.* In turn, the most relevant genes to explain the GBM cluster were the *CHI3L1, ANXA1, IGFBP3, COL1A2, COL1A1, NNMT, COL3A1, PDPN*, *POSTN, VEGFA, PTX3, TOP2A, HS3ST3B1, IGF2BP3, LOX, SHOX2, XIST* and *IBSP* genes. Of note, the *ETNPPL* gene among DA + AA tumors and the *CHI3L1* gene in GBM were those genes ranking the highest for each cluster. Interestingly, several of these differentially expressed genes identified in our study have also emerged as genes relevant for discriminating distinct subtypes of diffuse astrocytomas in other studies^12,15,23^, including the GBM-associated *CHI3L1*, *COL1A1*, *VEGFA* and *ANXA* genes (Supplementary Table S2). In turn, new gene associated here for the first time with DA + AA included the *DPP10* gene. The variability encountered among the 155 samples of the discovery cohort was then visualized in a low-dimensional space using canonical biplot representation.

Canonical biplot based on the 27 selected genes showed differential GEP for the samples visualized in a two-dimensional space (Fig. 2B). In this biplot representation most genes contributed substantially to the formation of the factorial horizontal axis 1, which provides a clear distinct structure between GBM and both DA + AA samples, the most discriminatory genes being the *ETNPPL, SH3GL2, GABRG2, SPX, DPP10, TOP2A, SHOX2, IGF2BP3, ANXA1, VEGFA* and *CHI3L1* genes. Some of these axis 1 genes showed higher expression values among DA + AA tumors (*ETNPPL, DPP10, SH3GL2, GABRG2, SPX)* while others were found to be overexpressed in GBM vs both DA + AA (e.g. *IGFBP3, IGF2BP3, SHOX2, VEGFA)*. In turn, vertical axis 2 was relevant to differentiate DA from AA gliomas. This later axis was mostly explained by differences in the expression levels of *XIST* together with differential expression levels of genes contributing both to axis 1 and 2, such as *SFRP2, FSTL5, TOP2A* and *SHOX2*, principally. Of note, *XIST* showed lower expression levels in DA tumors (versus some AA and GBM), while *FSTL5* and *SFRP2* expression was greater in DA vs AA gliomas. Similarly to NMF, canonical biplot confirmed the low contribution of *TOP2A* and *XIST* to the DA + AA cluster (and *SFRP2* to the GBM cluster), which was due to the fact that some AA samples had similar expression levels for both genes to those observed among GBM (or to the DA + AA cluster, in case of *SFRP2*) (Fig. 2B).

From the functional point of view, genes found to discriminate among the different subtypes of diffuse astrocytic gliomas corresponded to: (i) genes involved in development (*HOXD10, CNTN3, LOX, SFRP2, XIST*); (ii) cell adhesion (*IBSP*, *PDPN*, *POSTN*); (iii) cell metabolism (*ETNPPL, HS3ST3B1, NNMT, DPP10, SHOX2, SPX*); (iv) proliferation (*COL1A1, COL1A2, COL3A1, FSTL5, IGFBP3, IGF2BP3, TOP2A*); (v) angiogenesis (*VEGFA*); (vi) neurotransmission (*GABRB2, GABRG2, SH3GL2*); and (vii) the inflammatory response (*ANXA1*, *CHI3L1*, *PTX3*). Of note, DA was associated with greater expression of genes related to development and cell adhesion, AA to genes involved in metabolism and neurotrasmission and GBM to genes associated with angiogenesis and inflammatory responses (Fig. 2C).

**Figure 3:**
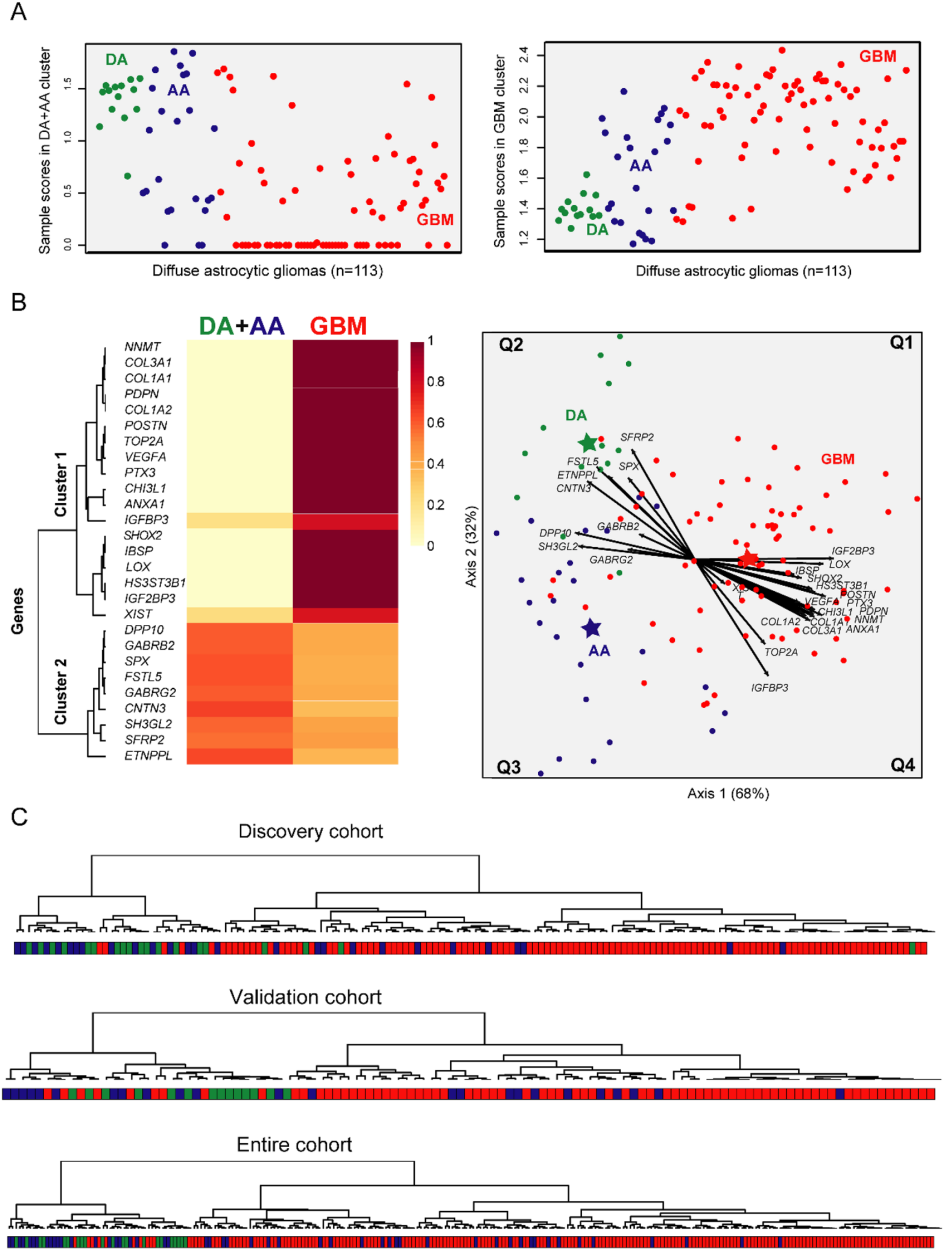
Multivariate analysis of the association between the 27 genes found to discriminate among different histopathological subtypes of diffuse gliomas in the validation set of 113 diffuse astrocytic gliomas. Nonnegative matrix factorization plots of contribution scores of each sample to the formation of each cluster (**A**) and the importance of each gene to the two clusters formed (**B**, left) are shown together with canonical biplot (**B,** right), that confirmed classification of diffuse astrocytic gliomas around two leading molecular groups: a group including mostly GBM tumors and another group mostly composed of both DA and AA tumors. Dendrograms obtained via non-hierarchical NMF clustering for each (discovery, validation and total cohort) sample are shown in panel **C.** For the distinction of sample scores in panel **A** see legend to Fig. 2**.** R software for statistical computing and graphics (v3.5.2) was used. DA, diffuse astrocytoma; AA, anaplastic astrocytoma; GBM, glioblastoma.

In order to confirm the discriminatory power of the combination of the 27 genes identified to discriminate among distinct tumor subtypes, validation in an independent cohort of 113 gliomas was performed (Fig. 3). In this validation series, NMF and canonical biplot clearly identified the same two clusters. The DA + AA cluster was mostly explained by the *SH3GL2, DPP10, GABRG2* and *GABRB2* genes, while the GBM cluster (including a few AA tumors) was characterized by the *VEGFA, POSTN, CHI3L1* and *SHOX2*, among other genes (Fig. 3A, B). *SFRP2, FSTL5* and *ETNPPL* were the genes mostly contributing to the separation between DA and AA. These series distinct GEP of different subtypes of diffuse astrocytic glioma that were also confirmed using unsupervised nonhierarchical cluster in discovery, validation and total cohorts (Fig. 3C). These results showed the same overall behavior with two clearly different clusters, where some AA samples showed the same genetic patterns as DAs, and others showed clear genetic similarities with GBM.

Finally, to quantify how well this same combination of 27 genes separates DA, AA and GBM samples, LDA analysis was performed for the validation cohort. Overall, LDA showed an accuracy of 87% (98/113 tumors were properly classified; 12/14 DA, 17/24 AA, 69/75 GBM) to predict for the tumor histopathological diagnosis based on GEP was observed (Fig. 3C). The remaining 15/113 (15%) tumors (2/14 DA, 7/24 AA and 6/75 GBM) were not accurately classified and they corresponded to: (i) 2 DA classified as AA; (ii) 1 AA classified as DA; (iii) 6 AA identified as GBM; (iv) 1 GBM classified as DA; and (v) 5 GBM classified as AA.

## Discussion

At present it is well established that multiple signaling pathways and cell functions are altered in astrocytic gliomas in association with a great number of recurrent genetic and microenvironmental changes^17^. In order to dissect the link between these genetic alterations and their functional consequences, GEP of DA, AA and GBM have been investigated in the last decade^12,14,16,24–27^. However, in only a few of these studies attempts have been made to identify genes differentially expressed between distinct histopathological glioma grades^14,24^, other studies focusing on the value of distinct GEP to help predicting tumor progression and patient survival^12,25^ or for identifying new targets for more effective therapy^27^. In turn, these studies have frequently been based on small patient cohorts^16,25^ with admixtures of tumors of all glioma subtypes^12,14,28^, they have used different microarray platforms^12,29,30^ and data analysis algorithms^12,31^, which have (frequently) led to controversial or even contradictory results.

Microarray GEP technologies provide massive data on the expression levels of thousand genes simultaneously but require the use of mathematical algorithms to capture the multidimensional nature of RNA expression data, in order to extract the critical information they might contain^31^. So far, these analyses have been based on distinct (frequently suboptimal) mathematical models which might introduce disturbing levels of variability on the study conclusions. Thus, some studies^16,31^ have used traditional multivariate statistical techniques, particularly PCA, which are not appropriate for data sets with few patients analyzed for thousands of variables, because of the variability overlooked in a high-dimensional space^32^. Because of these limitations of PCA and other traditional multivariate analysis algorithms, several new techniques have emerged for selection of the most informative variables under these circumstances^31^. Here we used CUR decomposition and univariate analysis (pairwise comparisons) to select for the most relevant genes to define diffuse astrocytic glioma subtypes in our discovery cohort. In order to further overcome analysis of samples that have a priori structured groups we used NMF, a method previously applied in glioma samples, which proved to provide useful conclusions to identify dynamic immune profiles during tumor evolution^17^. Based on this approach we identified unique GEP associated within the different histopathological subtypes of diffuse astrocytic gliomas. As expected, the largest GEP differences were found between DA and AA on one side, and GBM on the other side. In contrast, no differentially expressed genes were found to clear discriminate between DA and AA gliomas in the gene selection step (univariate analysis). Overall, these results are consistent with previous observations highlighting the remarkable difficulty to distinguish DA and AA based on mRNA GEP data^33^. Similarly, multivariate analysis based on NMF also revealed (only) two clearly different groups of diffuse astrocytic tumors, one including lower-grade astrocytomas (DA + AA) and the other GBM together with a few AA. Of note, these later AA might represent those AA tumors that might evolve to grade-IV glioma, further studies being required to confirm this hypothesis.

Lack of discrimination between DA and AA gliomas is not surprising and confirms that both tumor types display similar GEP and that they might potentially represent a single molecular subtype of astrocytomas. Even though, DA tumors showed lower expression of the *XIST* gene which together with overexpression of the *FSTL5* and *SFRP2* genes, once compared to AA tumors. Interestingly, *XIST* is a gene whose expression varies substantially with sex, that has been claimed to be a key gene in the oncogenesis of gliomas^34^. Since similar male/female distribution was observed in DA and AA tumors (data not shown) our results suggest that differences in *XIST* expression among DA and AA tumors might probably be due to its potential role on cell proliferation and invasion and requires further investigation. At the same time *FREM3* has been previously found to be overexpressed in both DA and AA vs oligodendroglioma tumors^35^, and *SFRP2* has been recently reported to contribute to the discrimination between DA and GBM^30^, in line with our findings. While *XIST* and *SFRP2* were the best discriminatory genes between DA and AA gliomas, additional differences between these tumor subtypes and GBM were found, including overexpression of the *DPP10*, *ETNPPL* and *SH3GL2* genes and underexpression of *CHI3L1, VEGFA* and *IGF* genes in the former two tumor types, in close association with unique chromosomal location profiles.

Overall, neither *IDH1/2*-mutations nor other chromosomal alterations showed the potential for being a discriminatory marker for distinct subtypes of diffuse astrocytomas in our own tumor cohort due to the low and variable *IDH*-mutational frequency and the heterogeneous cytogenetic profiles observed in GBM^36^ and other subtypes of diffuse astrocytomas, in line also with previous findings by others^37^. Thus, in the absence of other discriminatory markers, overexpression of *DDP10, ETNPPL* and *SH3GL2* in DA + AA vs GBM might be considered in distinguishing these tumor subgroups, particularly for unclassifiable tumors or in case of small biopsy samples. In line with previous findings for the *ETNPPL* gene^30^, this gene together with *SH3GL2*, and the *DPP10* gene identified here for the first time as relevant discriminatory gene, might represent a comprehensive panel of genes, mainly coded in chromosomes 4 and 2, to differentiate DA and AA gliomas. *DPP10* is a gene associated with cell development and inhibition of cell growth. Altogether, these findings further highlight the potential relevance of the loss of function of these genes during tumor progression to more advance diffuse astrocytic gliomas.

In this regard, GBM and some AA showed higher levels of expression of *TOP2A* compared to DA and to most AA, suggesting that this gene might be involved in determining a high proliferation rate among AA tumors, that might potentially progress to sGBM since gene is involved in promoting cell growth signals^38^.

Similarly, *IGF* genes, a family of genes previously associated with malignant astrocytomas^26,39^ and progression to sGBM^12,15^, were found here to progressively increase its expression from DA to AA and GBM. Of note, *IGFBP3* overexpression in GBM was associated in our study with overexpression of *VEGFA,* several collagen family genes (*COL1A1*, *COL1A2* and *COL3A1*)*,* and *CHI3L1,* among other genes. Since *VEGFA* interacts with *IGFBP2* during angiogenesis^40^, overexpression of *VEGFA* and *IGFBP3* genes might play an important role in tumor growth and expansion through promoting the formation of new blood vessels, in line with the increased angiogenesis observed in GBM^41^ vs other diffuse astrocytomas. In addition, the close association observed here between increased *VEGFA* and *COL1A2, COL3A1* and *COL1A1* expression in GBM, might also contribute to explain the effect of *VEGFA* on inducing collagenase expression and remodeling the tumor microenvironment in malignant astrocytomas (i.e. GBM). At the same time, these results support previous observations suggesting that *COL3A1* is a reliable biomarker of GBM^42^. Similarly, *CHI3L1,* a gene that encodes for a protein involved in the inflammatory response, found here to be associated with GBM, has been related to a poorer outcome of GBM, due to a greater invasion and shorter patient survival^23^.

Of note, the 27 gene panel here identified also showed a high accuracy to distinguish between the distinct histopathological subtypes of diffuse astrocytic tumors in our validation cohort. These results support its potential utility in the subclassification of diffuse astrocytic tumors in routine clinical practice, particularly for cases with inconclusive histopathological diagnosis. In addition, they might provide further prognostic information among AA. Further prospective studies in large series of astrocytoma patients are necessary to confirm our results and extend these findings to other subtypes of glioma, prior to their translation into routine laboratory diagnostics.

## Materials and methods

### Diffuse astrocytic tumor datasets

**Figure 4:**
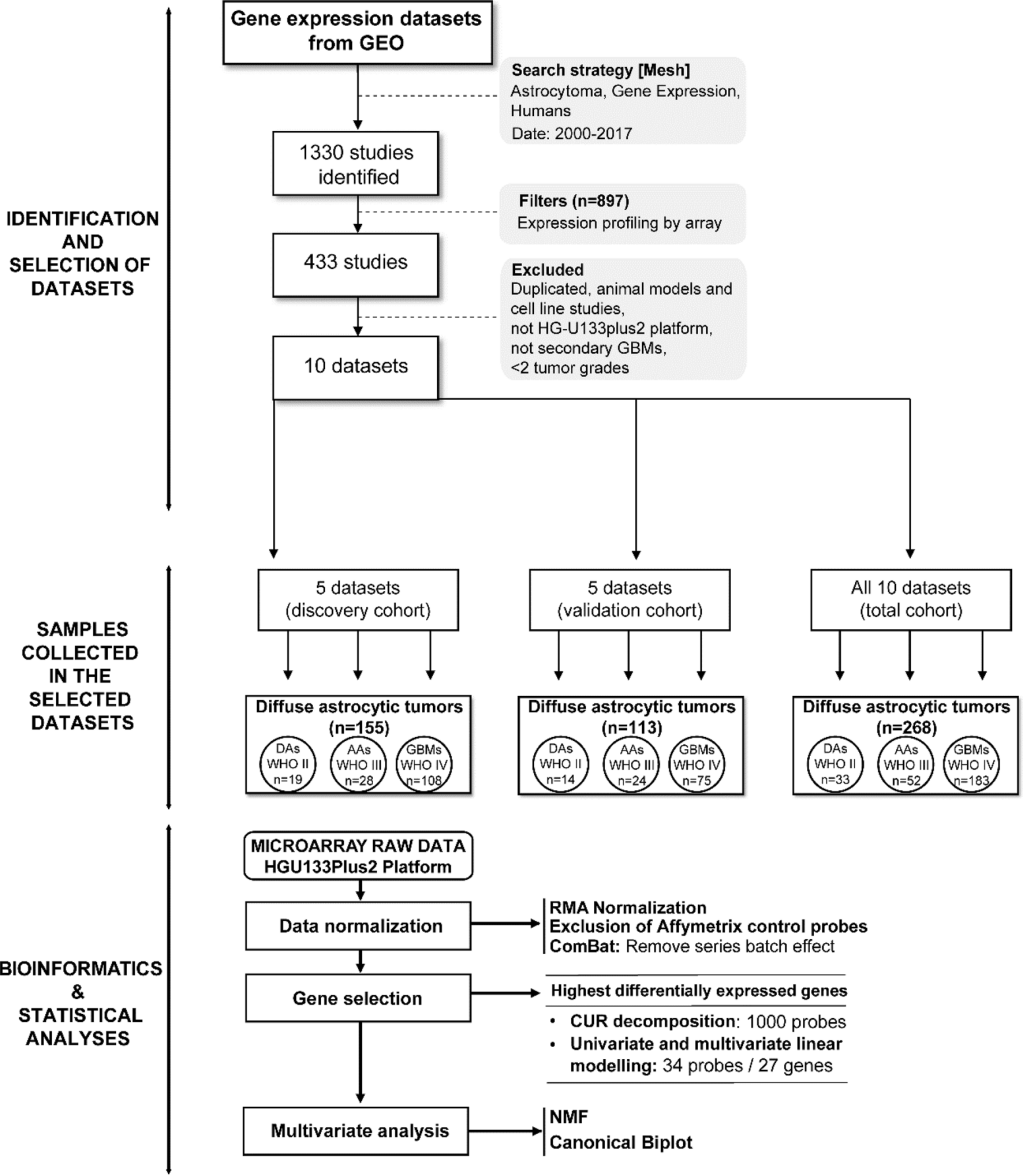
Flowchart summarizing sample data collection, selection criteria and steps, and the gene expression bioinformatics pipeline used in this study. Based on the filters and exclusion criteria, a total of 155 plus 113 diffuse astrocytic tumors grouped into 3 diffuse astrocytic tumor subtypes were included in the discovery and validation cohorts, respectively. For data analysis, individual variability was first removed by applying RMA, followed by the ComBat method. Then, those probes showing the highest differences among the groups of tumors were selected via CUR decomposition and pairwise comparisons. Based on the selected gene probes and multivariate analysis via nonnegative matrix factorization and canonical biplot graphical representation, those genes providing the highest discrimination among the distinct subtypes of astrocytic gliomas were identified.

Data from a total of 10 glioma patient cohorts was downloaded from the Gene Expression Omnibus (GEO, https://www.ncbi.nlm.nih.gov/geo/) public functional genomic repository (Fig. 4). The data search strategy included the “astrocytoma”, “gene expression” and “humans” medical subject headings (MeSH). Based on these terms, a total of 1,330 studies were identified between the year 2000 and 2017. From these studies, only those referred to “expression profiling by array” were further selected, resulting in 10 datasets that fulfilled the inclusion criteria: i) usage of the HG-U133Plus2 array platform for GEP analyses; and, ii) human tumor samples investigated. Cell line and animal model studies, as well as human tumor series containing secondary GBM and/or tumor samples of only one diffuse astrocytic histopathological grade were excluded from the analysis (Fig. 4). From the 10 series that fulfilled all inclusion criteria, 5 series (our own series^14^ and 4 additional cohorts^28,43–45^) consisting of a total of 155 diffuse gliomas were randomly selected to be used as discovery cohort with the following distribution according to the WHO-2016 diagnostic criteria: DA, 19 cases (11%); AA, 28 cases (16%) and GBM, 108 cases (73%) (Fig. 4; Supplementary Table S3). The remaining 5 patient series ^16,25,26,46^ were used for the validation cohort (Fig. 4; Supplementary Table S3), for a total of 113 diffuse astrocytic gliomas consisting of 14 DA (12%), 24 AA (21%) and 75 GBM (67%). The two (discovery and validation) cohorts also showed a similar distribution per age (median of 60 vs 56 years, respectively; p = 0.591, by the Mann–Whitney U) and sex (male/female ratio of 1.94 and of 1.63, respectively; p = 0.705 by the Fisher exact test) for patients for whom data on these features were publicly available (108/155 and 64/113 had data on age and 47/155 and 79/113 patients had data on sex, respectively).

### Normalization of gene expression data and data analysis tools

For data analysis summarized in Fig. 4, genomic tools from the Bioconductor and R environment for Statistical Computing and Graphics free software^47^ (v3.5.2; www.cran.r-project.org) were used. For data normalization, the robust multi-array average (RMA) expression measure was used. Subsequently, variability due to each GEO database was removed using the ComBat procedure included in the *sva* R-package, which shrinks the variance of independent studies^48^ initially detected by data analysis techniques. PCA score plots were used to visualize the stabilized series patterns (Supplementary Fig. S1A). Gene symbols of the 54,675 probes were annotated and those without associated information, as well as those corresponding to Affymetrix control probes, were excluded from further analyses. Multiple probes of the same gene were kept in the analysis in order to minimize loss of differences between glioma subtypes. Finally, a total of 44,723 probe sets corresponding to RNA expression probes of 21,336 genes were included in the analysis (Supplementary Table S4). Bioinformatics analyses were implemented using the R-package *rCUR* to perform CUR decomposition^49^, *limma* to detect significant genes with differential gene expression levels between groups -providing models to compare many RNA targets simultaneously^50^, *NMF* to conduct NMF factorization^19^ and *MultBiplotR* to perform Canonical Biplot analysis and representation^51^. The R-code to reproduce the analyses in this work is available in https://github.com/ananieto/Scientif-Reports-Gonzalez-Garcia_et_al.

### Gene selection

Data analysis for selection of differentially expressed genes was performed in two sequential steps. First, the 1,000 probes showing the greatest variability were selected based on factors of influence (called leverages) of CUR decomposition^21^ (Fig. Supplementary S1B). Subsequently, supervised analysis was performed to identify differentially expressed genes, based on FC values. For this purpose, univariate linear modelling was first performed to identify those gene probes that showed significant differences in expression levels between different astrocytic tumor subtypes in pairwise comparisons (i.e. DA vs AA, DA vs GBM, AA vs GBM). P-values obtained were adjusted by applying the Benjamin-Hochberg correction -also termed BH or False Discovery Rate (FDR) as the most widely used for genomics studies. FDR controls the expected value for the proportion of false positive cases among the null hypotheses rejected. Those genes with significant adjusted p-values which also differed in gene expression levels by FC ≥ 4 between the tumor grade groups, were selected.

### CUR decomposition

CUR is defined as a low-range approximation of a matrix ${{\varvec{X}}}_{I x J },$ expressed in a small subset of rows and/or columns^21^. In this work, we select the variables (i.e. probes) that mostly contributed to the model in terms of variability. Leverage is defined as the amount of variance contributed by each variable to the factorial model, similar to the explanatory power of a variable in regression analysis. Since our goal was to select the probes with greater variability, for each of them we defined their leverage (${l}_{j}$) as

$${l}_{j}=\frac{1}{K}\sum_{r=1}^{R}{({v}_{jk})}^{2}, j \epsilon \{1,\dots ,44723\}$$

where ${v}_{j} (j=1,\dots ,44723)$ is the right singular vectors obtained in Singular Value Decomposition (SVD) of $X$, and $R$ the number of latent variables in the dimension reduction process. In our analysis, $R$ was equal to the number of PCs needed to absorb all the variability.

### Multivariate analysis based on NMF and canonical biplot representation

All differentially expressed gene probes displaying FC > 4 in pairwise comparisons were subsequently included in multivariate analysis based on NMF^19^. Briefly, NMF is an unsupervised clustering method defined as a matrix factorization technique that decomposes the original dataset ($X\in {\mathbb{R}}_{+}^{J x I}$) into two positive matrices whose product closely approximates $X (X\approx WH$, where $I$ and $J$ refer to the number of samples and genes, respectively, $W\in {\mathbb{R}}_{+}^{J x K}$ and $H\in {\mathbb{R}}_{+}^{K x I}$ are nonnegative coefficients matrices and $K$ is the number of clusters retained). The $H$ matrix can be used to group the *I* samples into *K* clusters. Its columns represent the membership of each sample to the clusters. $W$ rows define the physical meaning of clusters in terms of gene expression, where an ${w}_{jk}$ element symbolizes the expression level of gene $j$ in cluster $k$; i.e., $W$ denotes the contribution of each gen to the cluster, in such a way that the higher the contribution, the more important that gene is in the formation of its cluster. Finally, canonical biplot^22^ was used as a visualization tool of the multivariate data matrix. For this purpose, a priori structure of groups in a low dimensional space with maximum discriminatory power between classes was used, in which the discriminatory genes involved in separation of the three histological tumor subtypes are shown. To facilitate its interpretation, an example is provided in Supplementary Figure S2. In order to validate the power of the contribution of the discriminatory genes selected above through NMF analysis, to classify individual samples into the distinct WHO 2016 tumor subtypes, unsupervised NMF was applied to the discovery cohort (n = 155), the validation set (n = 113) and the entire cohort (n = 268) of diffuse astrocytic gliomas.

### Linear discriminant analysis (LDA)

To validate the power of the contribution of the discriminatory genes selected above through NMF analysis and quantify how well the gene signature separates DA and AA gliomas, LDA was applied to the validation set of 113 diffuse astrocytic gliomas and the percentage of correct tumor classification recorded.

## Supplementary information

Supplementary Dataset

Supplementary Figures

Supplementary Table S1, S2, S3

Supplementary Table S4

## Data Availability

The datasets analysed during the current study are available in the GEO repository, https://www.ncbi.nlm.nih.gov/geo/.
