# Supplementary material for: Multivariate analysis reveals differentially expressed genes among distinct subtypes of diffuse astrocytic gliomas: diagnostic implications
Source: Sci Rep. 2020 Jul 9;10:11270. doi: 10.1038/s41598-020-67743-7 (PMC7347847; doi:10.1038/s41598-020-67743-7)
Supplement: Supplementary file 2 — Supplementary Figures [file 41598_2020_67743_MOESM2_ESM.pdf]

Title: Multivariate analysis reveals differentially expressed genes among distinct subtypes of diffuse astrocytic gliomas: diagnostic implications.

Authors: Nerea González-García[1,2], Ana Belén Nieto-Librero[1,2], Ana Luisa Vital[3], Herminio José Tao[4], María González-Tablas[2,5,6], María Purificación Galindo-Villardón[1,2], Alberto Orfao[2,5,6], María Dolores Tabernero [2,5,6,7].

Institutions: (1) Department of Statistics, University of Salamanca, Spain; (2) Instituto de Investigación biomédica de Salamanca, IBSAL- University Hospital of Salamanca, Salamanca, Spain; (3) Centre for Neuroscience and Cell Biology and Faculty of Pharmacy, University of Coimbra, Portugal; (4) Neurosurgery Service, University Hospital of Coimbra, Portugal, Coimbra; (5) Centre for Cancer Research (CIC-IBMCC; CSIC/USAL; IBSAL) and Department of Medicine, University of Salamanca, Salamanca, Spain; (6) Biomedical Research Networking Centre on Cancer–CIBERONC (CB16/12/00400), Institute of Health Carlos III, Madrid, Spain; (7) Instituto de Estudios de Ciencias de la Salud de Castilla y León (IECSCYL-IBSAL), Salamanca, Spain

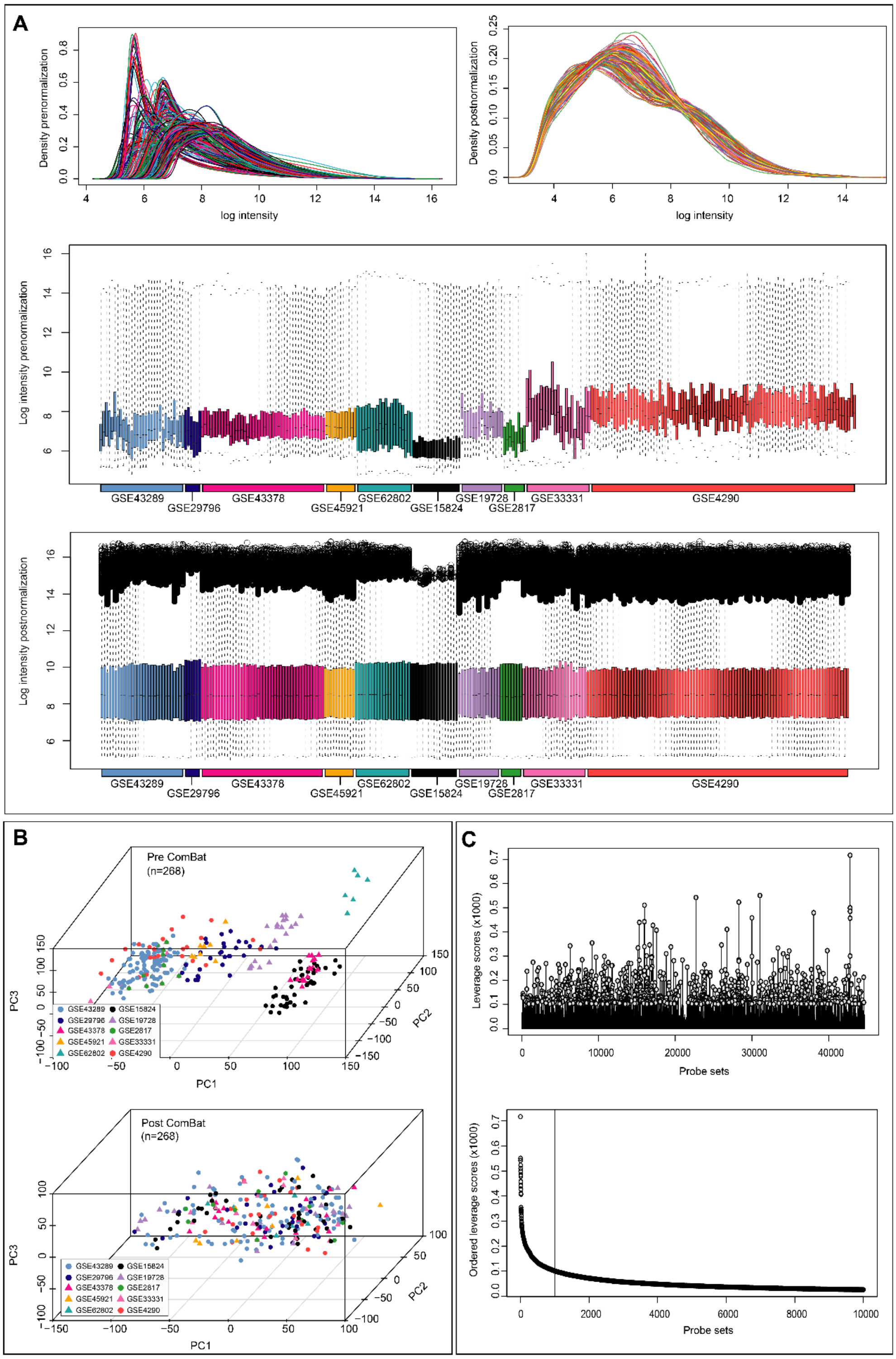

Supplementary Figure S1. Gene expression normalization of astrocytic tumor data. Density plots and boxplots for all samples in the discovery and validation cohorts prior to and after RMA normalization (**A**). PCA score plot illustrating data variability before and after implementation of the ComBat method to remove the specific variability of individual samples from the 5 tumor series in the discovery cohort analyzed (n = 155 astrocytic tumors, 44,723 probes) (**B**) and probe variability leverages provided by CUR decomposition, followed by an ordered list of genes with decreasing variability (**C**).

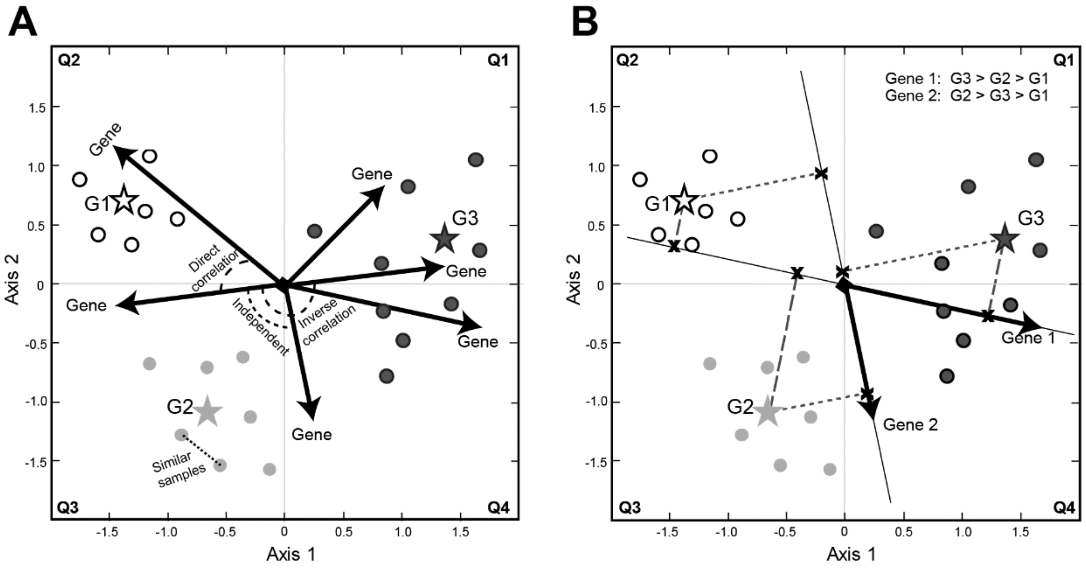

Supplementary Figure S2. Canonical biplot interpretation of multivariate data matrix to visualize differences among three distinct tumor subtypes (G1, G2 and G3). Distances between points reflect the similarity between samples, stars represent the mean value of each tumor subtype and arrows (vectors) represent the discriminatory genes (**A**). The length of each vector reflects gene expression variability and the cosines of the angles between vectors reflect the correlation that exists between them (acute angles reflect a direct relationship between genes, while obtuse angles reflect inversely correlated genes). In B, differences in gene expression levels are shown (the mean expression value of gene 1 and gene 2) for different groups of samples plotted via the perpendicular projection from the corresponding star onto the gene (direction of the vector), where the closer the tumor is to the arrowhead, the higher the gene expression value is. Thus, gene 1 has the highest expression in G3, followed by G2 and G1, and gene 2 has higher expression in G2 samples, followed by G3 and G1 samples.
