# Supplementary material for: Multivariate analysis reveals differentially expressed genes among distinct subtypes of diffuse astrocytic gliomas: diagnostic implications
Source: Sci Rep. 2020 Jul 9;10:11270. doi: 10.1038/s41598-020-67743-7 (PMC7347847; doi:10.1038/s41598-020-67743-7)
Supplement: Supplementary file 3 — Supplementary Table S1, S2, S3 [file 41598_2020_67743_MOESM3_ESM.pdf]

Title: Multivariate analysis reveals differentially expressed genes among distinct subtypes of diffuse astrocytic gliomas: diagnostic implications.

Authors: Nerea González-García[1,2], Ana Belén Nieto-Librero[1,2], Ana Luisa Vital[3], Herminio José Tao[4], María González-Tablas[2,5,6], María Purificación Galindo-Villardón[1,2], Alberto Orfao[2,5,6], María Dolores Tabernero [2,5,6,7].

Institutions: (1) Department of Statistics, University of Salamanca, Spain; (2) Instituto de Investigación biomédica de Salamanca, IBSAL- University Hospital of Salamanca, Salamanca, Spain; (3) Centre for Neuroscience and Cell Biology and Faculty of Pharmacy, University of Coimbra, Portugal; (4) Neurosurgery Service, University Hospital of Coimbra, Portugal, Coimbra; (5) Centre for Cancer Research (CIC-IBMCC; CSIC/USAL; IBSAL) and Department of Medicine, University of Salamanca, Salamanca, Spain; (6) Biomedical Research Networking Centre on Cancer–CIBERONC (CB16/12/00400), Institute of Health Carlos III, Madrid, Spain; (7) Instituto de Estudios de Ciencias de la Salud de Castilla y León (IECSCYL-IBSAL), Salamanca, Spain

Supplementary Table S1. Gene symbols, full names and major functions of the 26 genes differentially expressed in distinct subtypes of diffuse astrocytic gliomas

Gene symbol	Gene name	Gene function
ANXA1	Annexin A1	Regulator of inflammatory process
CHI3L1	Chitinase 3 like 1	Polysaccharide binding
CNTN3	Contactin 3	Cell adhesion and neural development
COL1A1	Collagen type I alpha 1 chain	Platelet derived growth factor binding
COL1A2	Collagen type I alpha 2 chain	Platelet derived growth factor binding
COL3A1	Collagen type III alpha 1 chain	Platelet derived growth factor binding
DPP10	Dipeptidyl peptidase like 10	Voltage potassium channels binding
ETNPPL	Ethanolamine-phosphate phospho-lyase	Ethanolamine-phosphate phospho-lyase activity
FSTL5	Follistatin like 5	Calcium binding. Anti-proliferative activity
GABRB2	Gamma-aminobutyric acid type A receptor beta2 subunit	Component of GABA receptor. Neurotrasmission
GABRG2	Gamma-aminobutyric acid type A receptor gamma2 subunit	Component of GABA receptor. Neurotrasmission
HS3ST3B1	Heparan sulfate-glucosamine3-sulfotransferase 3B1	Heparan sulfate process
IBSP	Integrin binding sialoprotein	Cell-matrix interaction in bone microenviroment
IGF2BP3	Insulin like growth factor 2 mrna binding protein 3	Translation of insulin like growth factor binding
IGFBP3	Insulin like growth factor binding protein 3	Translation of insulin like growth factor binding
LOX	Lysyl oxidase	Oxidoreductase
NNMT	Nicotinamide N-methyltransferase	Nicotinamide N-methyltransferase activity
PDPN	Podoplanin	Adhesion and migration protein
POSTN	Periostin	Adhesion and migration of epithelial cells
PTX3	Pentraxin 3	Inflammation and component activation regulator
SFRP2	Secreted frizzled related protein 2	Wnt signaling modulation
SH3GL2	SH3 domain containing GRB2 like2	Protein binding. Neurotrasmission-associated
SHOX2	Short stature homeobox 2	Transcriptional regulator
SPX	Spexin hormone	Galanin receptors ligand
TOP2A	Topoisomerase DNA II alpha	DNA binding
VEGFA	Vascular endothelial growth factor A	Proliferation and migration of vascular endothelial cells
XIST	X inactive specific transcript	Chromosome X inactivation

Supplementary Table S2. Genes differentially expressed in distinct subtypes of diffuse astrocytic gliomas identified in our study (500 DEGP) in common to previous reports on >10 diffuse astrocytic tumors, including DA, AA and GBM (5 series) or AA and GBM (4 series)

Genes in common	Series	Previous series N. of samples analyzed Total	DA	AA	GBM	Probe codes	Mean FC values DA	AA	GBM
ANXA1	GSE54004[a]	143	12	33	98	201012_at	8.9	9.6	11.3
CHI3L1						209396_s_at<br>209395_at	8.1	9.2	12.2
COL1A1						1556499_s_at<br>202310_s_at<br>202311_s_at	6.8	7	9.1
COL1A2						202404_s_at	7.1	7.5	10
GPR17						206190_at<br>215225_s_at	7.6	7.5	6.5
MMP9						203936_s_at	7	7	8.1
VEGFA						211527_x_at	6.7	7.6	10
GPNMB	GSE43378[b]	50*	5	7	32	201141_at	8.8	8.5	9.9
EPHB1	GSE19728[c]	15	5	5	5	230425_at	9.2	9	8.1
STXBP6	GSE43289[d]	40*	2	2	26	230560_at	8.1	7.8	5.8
TOP2A						201291_s_at	6.1	7.0	8.6
ADM	GSE1993[e]	65*	5	19	39	202912_at	7.7	7.7	10.1
FABP5						202345_s_at	9.5	9.6	11
PDGFRA						203131_at	11	10.9	10.1
FBXO32	GSE2223[f]	50*	-	1	27	241762_at	6	5.9	6.9
IGFBP2						202718_at	7.2	8.4	11
SOD2						215078_at	4.9	5	6.4
CCL2	GSE33331[g]	26*	-	2	21	216598_s_at	9	8.6	9.8
GBP1						231577_s_at<br>202269_x_at<br>202270_at	7.9	7.9	9.5
GBP2						242907_at	7.3	7.3	8.7
IGHM						209374_s_at	6.2	5.8	6.3
SAA2						214456_x_at	6.6	6.9	7.9
XAF1						228617_at	8.1	8.5	9.2
CHI3L1	GSE4271[h]	91	-	24	77	209396_s_at	8.1	9.2	12.2

DLL3						219537_x_at<br>222898_s_at	8.5	8.6	7.4
TOP2A						201291_s_at	6.1	7	8.6
CALCRL						210815_s_at	8.7	8.3	7.7
COL6A3						201438_at	6.7	6.7	8.4
DLL3						219537_x_at	8.5	8.6	7.4
IGFBP2						202718_at	7.2	8.4	11
LOX						215446_s_at<br>204298_s_at	5.1	5.4	7.5
MET						203510_at	6.8	6.6	7.5
MGP						202291_s_at	8.3	8.3	9.7
MMP9	GSE4412[i]	74	-	24	50	203936_s_at	7	7	8.1
RGS4						204337_at<br>204338_s_at	8.7	8.3	7.1
SYT1						203999_at<br>203998_s_at	9.3	9	7
THBS1						201110_s_at	5.6	5.2	7
VSNL1						203798_s_at<br>203797_at	9.7	9.3	7.3

*: series including pilocytic astrocytomas, oligodendrogliomas or mixed tumors. [a]: Xu, Y. *et al.* Screening critical genes associated with malignant glioma using bioinformatics analysis. *Mol. Med. Rep.* **16**, 6580–6589 (2017).[b]: Kawaguchi, A. *et al.* Gene expression signature-based prognostic risk score in patients with glioblastoma. *Cancer Sci.* **104**, 1205–1210 (2013). [c]: Liu, Z., Yao, Z., Li, C., Lu, Y. & Gao, C. Gene expression profiling in human high-grade astrocytomas. *Comp. Funct. Genomics* **2011**, 245137 (2011). [d]: Vital, A. L. *et al.* Gene expression profiles of human glioblastomas are associated with both tumor cytogenetics and histopathology. *Neuro. Oncol.* **12**, 991–1003 (2010). [e]: Petalidis L. P. *et al.* Improved grading and survival prediction of human astrocytic brain tumors by artificial neural network analysis of gene expression microarray data. *Mol Cancer Ther* **7**, 1013-24 (2008). [f]: Bredel M. *et al.* Tumor Necrosis Factor-α–Induced Protein 3 As a Putative Regulator of Nuclear Factor-κB–Mediated Resistance to O6-Alkylating Agents in Human Glioblastomas. *J Clin Oncol* **24**, 274-287 (2006). [g]: Donson, A. M. *et al.* Increased immune gene expression and immune cell infiltration in high-grade astrocytoma distinguish long-term from short-term survivors. *J. Immunol.* **189**, 1920–7 (2012). [h]: Phillips, H. S. *et al.* Molecular subclasses of high-grade glioma predict prognosis, delineate a pattern of disease progression, and resemble stages in neurogenesis. *Cancer Cell* **9**, 157–173 (2006). [i]: Freije W.A. et al. Gene expression profiling of gliomas strongly predicts survival. *Cancer Res* 64, 6503-10 (2004).

Supplementary Table S3 Diffuse astrocytic glioma discovery series (n = 155), validation cohort (n = 113) and test sets (n = 268) analyzed using the HGU133Plus2 array (54,675 probes for 21,336 analyzed genes) deposited in the GEO repository and included in the present study

Accession ID	WHO 2016 diagnostic subtype				Reference
	Total (n=268)	DA (n=33)	AA (n=52)	GBM (n=183)	
Discovery series	155	19	28	108	
GSE4290	94	7	19	68	Sun et al. [43]
GSE43289	30	2	2	26	Vital et al. [14]
GSE15824	17	4	3	10	Grzmil et al. [28]
GSE2817	8	5	3	-	Turkheimer et al. [44]
GSE29796	6	1	1	4	Auvergne et al. [45]
Validation series	113	14	24	75	
GSE43378	44	5	7	32	Kawaguchi et al. [25]
GSE62802	20	-	7	13	(Not published)
GSE45921	11	4	3	4	Zhou et al. [46]
GSE19728	15	5	5	5	Liu et al. [16]
GSE33331	23	-	2	21	Donson et al. [26]
Test series	268	33	52	183	

DA: diffuse astrocytoma. AA: anaplastic astrocytoma. GBM: glioblastoma multiforme. ID: Specific code for each dataset in the GEO database.
